# Aurora kinase A interacts with H-Ras and potentiates Ras-MAPK signaling

**DOI:** 10.18632/oncotarget.15049

**Published:** 2017-02-03

**Authors:** MaKendra Umstead, Jinglin Xiong, Qi Qi, Yuhong Du, Haian Fu

**Affiliations:** ^1^ Graduate Program in Cancer Biology, Emory University, Atlanta, GA, USA; ^2^ Department of Pharmacology and Emory Chemical Biology Discovery Center, Emory University School of Medicine, Atlanta, GA, USA; ^3^ Department of Dermatology, Xiangya Hospital, Central South University, Changsha, China; ^4^ Winship Cancer Institute, Atlanta, GA, USA

**Keywords:** Aurora A, Ras, Raf, MAPK, protein-protein interactions

## Abstract

In cancer, upregulated Ras promotes cellular transformation and proliferation in part through activation of oncogenic Ras-MAPK signaling. While directly inhibiting Ras has proven challenging, new insights into Ras regulation through protein-protein interactions may offer unique opportunities for therapeutic intervention. Here we report the identification and validation of Aurora kinase A (Aurora A) as a novel Ras binding protein. We demonstrate that the kinase domain of Aurora A mediates the interaction with the N-terminal domain of H-Ras. Further more, the interaction of Aurora A and H-Ras exists in a protein complex with Raf-1. We show that binding of H-Ras to Raf-1 and subsequent MAPK signaling is enhanced by Aurora A, and requires active H-Ras. Thus, the functional linkage between Aurora A and the H-Ras/Raf-1 protein complex may provide a mechanism for Aurora A's oncogenic activity through direct activation of the Ras/MAPK pathway.

## INTRODUCTION

The Ras family of proteins (H-, K-, and N-Ras) are well characterized oncogenic drivers in a variety of cancer types [1, 2]. Ras proteins are GTPases, which function as molecular switches to regulate molecular signaling cascades in response to extracellular signals. Ras binds to and hydrolyzes GTP, cycling between active, GTP-bound and inactive, GDP-bound states [3, 4]. Structurally, the switch I and II domains of Ras also change in conformation when either GTP or GDP is bound [5]. Ras activity is facilitated by GTPase-activating proteins (GAPs) that facilitate hydrolysis of GTP to GDP, thereby returning Ras to an inactive state [6–8]. Conversely, guanine nucleotide exchange factor proteins (GEFs) bind to GDP-bound Ras, and help to exchange GDP for GTP and activate Ras. Point mutations in critical regions of Ras also affect activity. The oncogenic Ras G12V mutation prevents hydrolysis of GTP, locking the protein in an active conformation [9–11]. This mutation is commonly observed in patients and is known to drive cancer progression. In contrast, the dominant negative Ras S17N mutant inhibits RasGEF activity, maintaining its inactive Ras conformation [12].

Activated Ras recruits distinct effector proteins to initiate cellular signaling cascades and physiological processes [13–15]. Specifically, Ras sustains pro-growth and proliferative signaling through activation of the Ras-Raf-MEK-ERK (mitogen activated protein kinase, MAPK) pathway [16]. Hyperactive Ras-MAPK signaling increases the transcription of genes that drive the cellular growth and survival required for cancer progression [17].

Ras-MAPK signaling can upregulate transcription of the mitotic kinase, Aurora kinase A (Aurora A) [18]. Aurora A belongs to a family of serine/threonine kinases (Aurora A, B, and C) that function in different spatial and temporal points in the cell to facilitate mitosis [19, 20]. Aurora A expression is upregulated during mitosis where it facilitates alignment of microtubules to the centromeres, then is quickly degraded during mitotic exit [21, 22]. Amplification of Aurora A occurs at the DNA, RNA, and protein levels in several cancer types, such as breast, glioblastoma, pancreatic, and bladder cancers [23–26]. Recent literature has revealed details about the physiological impact of Aurora A overexpression beyond its canonical role in the cell cycle. For example, Aurora A can aid in oncogenic processes through forming different protein-protein interactions with many proteins, including GTPases [27, 28]. This may be facilitated by the observed mis-localization of Aurora A to both the cytoplasm and nuclear compartments in tumor tissue [29, 30]. Clinical data further support the role of Aurora A in cancer as overexpression of Aurora A is correlated with a worse prognosis and patient outcome [30, 31].

Interestingly, literature provides evidence of cooperation between Ras-MAPK signaling and Aurora A beyond transcriptional regulation. Aurora A amplification co-occurs in several cancer types with deregulated Ras signaling [32–35]. Also, Aurora A can enhance the transformation of fibroblasts harboring activating Ras mutations, while knock-down of Aurora A correlates to decreased MAPK signaling [36]. Although these studies point towards a function for Aurora A upstream of MAPK signaling, how Aurora A engages the MAPK pathway is critical to further elucidate its role as an oncogene in cancer.

Here we report that the protein-protein interaction of Aurora A and H-Ras is a mechanism by which Aurora A functions upstream of H-Ras to promote MAPK signaling. The Aurora A and H-Ras interaction validated in this study provides a novel link and potential positive feedback loop between two oncogenic proteins known to drive proliferation and survival in cancer. Blocking this interaction may have promising therapeutic potential to inhibit Ras-MAPK activity in cancer.

## RESULTS

### Aurora A is a novel H-Ras binding partner

**Figure 1:**
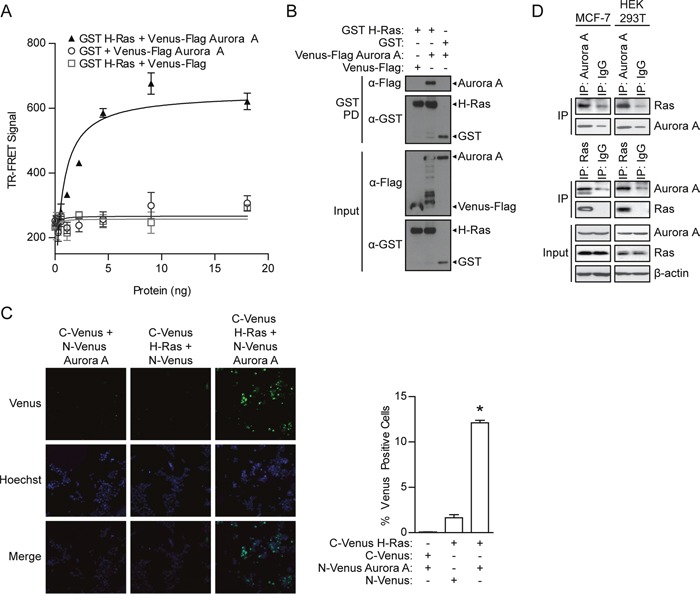
Detection of the Aurora A/H-Ras interaction **A**. TR-FRET assay performed using lysates from HEK 293T cells in which GST H-Ras was co-expressed with Venus-Flag Aurora A or vector controls. TR-FRET signal calculated as X/Y*Z; Tb ex 340 nm; Tb em 486 nm (X); Venus em 520 nm (Y); Z = 10^4^). TR-FRET signals were recorded using an EnVision multilabel plate reader. Data shown are average signals with SD from duplicate samples. **B**. GST pull-down assay conducted after GST H-Ras complexes were isolated from HEK 293T cell lysates with co-expressed Venus-Flag Aurora or appropriate controls. The presence of Venus-Flag Aurora A in the GST H-Ras protein complex (GST PD) and protein expression levels in the cell lysate (Input) was detected by Western blotting using anti-Flag or anti-GST antibody, respectively. **C**. A Venus protein-fragment complementation (Venus PCA) assay was conducted in living HEK 293T cells co-expressing N-Venus Aurora A and C-Venus H-Ras or vector controls. Interaction between tagged proteins allowed reconstitution of fluorescent Venus protein. The percentage of Venus positive cells was quantified by fluorescence imaging and scoring from two independent experiments. The percentage represents the number of cells with positive interactions compared to the total number of cells (determined by Hoechst staining). Representative images: Venus (positive protein-protein interaction), Hoechst (nucleus), Merge (overlap of Venus and Hoechst signals). **D**. Co-immunoprecipitation assay performed using lysates from HEK 293T and MCF7 cells. The Aurora A/Ras interactions are shown in both directions with IP-Aurora A and IP-Ras.

To gain insight into Aurora A signaling pathways and oncogenic activities, we tested whether Aurora A directly interacted with a variety of signaling proteins, including Ras. We first used the homogeneous, solution-based time-resolved Föster resonance energy transfer (TR-FRET) assay to detect binding [37]. The assay has a stringent distance requirement (<10 nm) between two interacting partners for the generation of TR-FRET signals. Therefore, TR-FRET signals in this assay format indicate the interaction between two proteins. To monitor the interaction of Aurora A and H-Ras, TR-FRET was performed using HEK 293T cell lysate with co-expressed GST H-Ras and Venus-Flag Aurora A. Co-expression of GST H-Ras and Venus-Flag Aurora A led to the generation of TR-FRET signals in a dose-dependent manner (Figure 1A). As background controls, no TR-FRET signal was detected with GST H-Ras or Venus-Flag Aurora A expression alone. Such a specific increase of the TR-FRET signal supports the direct interaction between Aurora A and H-Ras.

To confirm the Aurora A/H-Ras interaction detected by TR-FRET, a GST pull-down was performed as a secondary affinity-based binding assay. GST pull-downs were conducted with lysates from HEK 293T cells co-expressed with GST H-Ras with Venus-Flag Aurora A. Aurora A was found to pull down with GST H-Ras complex, but not in control lanes with GST (Figure 1B), demonstrating the association of Aurora A with H-Ras and confirming the previous TR-FRET results.

TR-FRET and GST pull-down assays are both *in vitro* cell lysate-based assays, thus, we further validated the interaction of Aurora A with H-Ras *in vivo* by utilizing a fluorescence (Venus)-based protein-fragment complementation assay (PCA). In this assay, N-Venus or C-Venus fragments are fused to two interacting proteins. The association of these proteins leads to functional reconstitution of Venus and allows the detection of green fluorescence signal using imaging. For this purpose, Aurora A and H-Ras were fused with N-Venus and C-Venus, respectively, and co-expressed in HEK 293T cells. The percentage of cells with positive protein-protein interactions (reconstituted Venus) was revealed by fluorescence imaging. Co-expression with N-Venus or C-Venus established background (Figure 1C). Co-expression of N-Venus Aurora A and C-Venus H-Ras resulted in an increase in the number of fluorescent cells compared to the expression of N-Venus Aurora A or C-Venus H-Ras with negative controls. Reconstitution of the Venus signal resulting from the interaction of Aurora A and H-Ras validates the presence of the interaction in living cells. The interaction was also detected in Cos7 fibroblast cells, MCF7 breast cancer cells, and 8-MG-BA glioblastoma cells (data not shown).

Finally, to test the interaction of endogenous Aurora A and H-Ras, co-immunoprecipitation was conducted using lysates from HEK 293T cells and the human breast cancer cell line, MCF7. Aurora A and Ras were isolated from cells using an Aurora A or pan-Ras antibody, respectively, but not an IgG control antibody (Figure 1D). Western blotting confirmed the presence of Ras in the Aurora A immunocomplex. Similarly, Aurora A was detected in the Ras immunocomplex, providing additional evidence for the Aurora A/H-Ras interaction at endogenous protein levels.

Overall, the Aurora A/H-Ras interaction was confirmed by four complementary approaches for monitoring protein-protein interactions, supporting Aurora A as a binding partner of H-Ras. Thus, the binding of Aurora A and H-Ras may provide a new mechanism for Ras regulation.

### Aurora A interacts with H-Ras through the switch I and II regions

**Figure 2:**
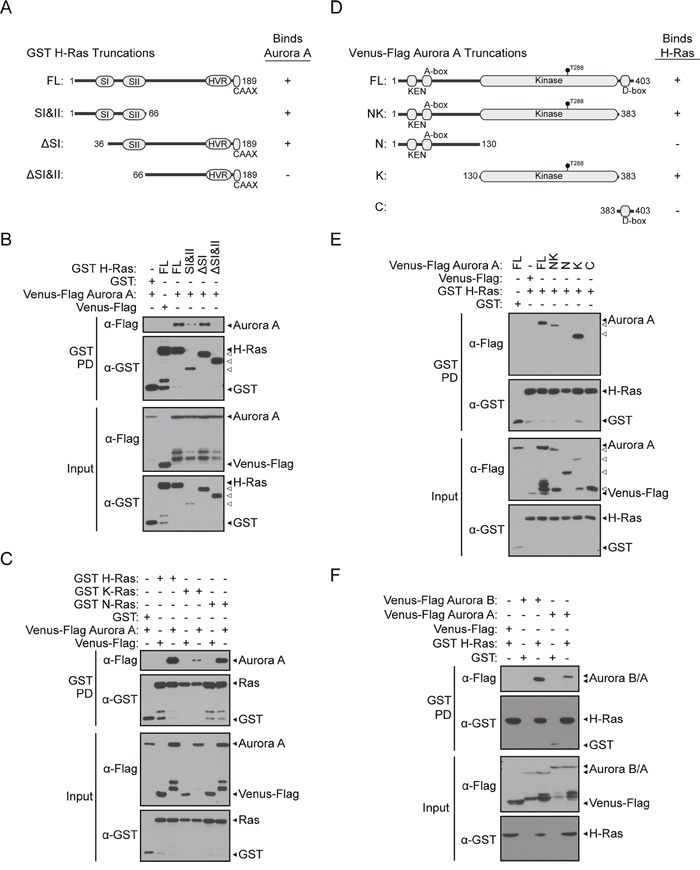
Interactions between Aurora A/B and Ras proteins are mediated through conserved domains **A**. Diagram of GST H-Ras protein domains and truncations used for deletion analysis: FL (amino acids 1-189), SI&II (amino acids 1-66), ΔSI (amino acids 36-189), ΔSI&II (amino acids 66-189). **B**. Characterization of the H-Ras protein domain responsible for binding to Aurora A. GST pull-down conducted from HEK 293T cells co-expressing GST H-Ras truncations and Venus-Flag Aurora A. Western blotting using anti-Flag or anti-GST antibody allowed detection of GST H-Ras peptides that were able to isolate full-length Aurora A. Full-length Aurora A/H-Ras protein binding was used as a positive control. **C**. Aurora A exists in protein complexes with H-, K-, or N-Ras. Binding of Aurora A as detected in GST pull-downs conducted from HEK 293T cells expressing GST H-Ras, GST K-Ras, or GST N-Ras and Venus-Flag Aurora A along with vector controls. **D**. Characterization of the H-Ras binding domain on Aurora A. Diagram of Aurora A protein domains and truncations used for deletion analysis: FL (amino acids 1-403), NK (amino acids 1-383), N (amino acids 1-130), K (amino acids 130-383), C (amino acids 383-403). **E**. GST pull-down conducted from HEK 293T cells co-expressing full-length GST H-Ras and Venus-Flag Aurora A truncations and analyzed by western blotting. Binding between full-length proteins served as a positive control. **F**. Aurora B interacts with H-Ras. Like Aurora A, Aurora B can be isolated in a protein complex with H-Ras. Binding of Aurora B as detected in GST pull-downs conducted from HEK 293T cells expressing GST H-Ras and Venus-Flag Aurora B along with vector controls was identified by western blotting using anti-Flag or anti-GST antibodies.

Ras proteins contain several key conserved regions that are involved in protein binding and oncogenic activity. To further characterize the Aurora A/H-Ras interaction, we next determined the structural domains that mediate binding using deletion analysis coupled with GST pull-downs. H-Ras truncations were generated and tested for their ability to bind Aurora A. The GST H-Ras truncations tested for binding are shown in Figure 2A: a region that includes the switch I and II domains (SI&II, amino acids 1-66), deletion of the switch I domain (ΔSI, amino acids 36-189), deletion of the switch I and II domains (ΔSI&II, amino acids 66-189). Our results show that when co-expressed in HEK 293T cells, binding of Aurora A was detected with full-length H-Ras but not with GST (Figure 2B). Aurora A was detected in complex with H-Ras SI&II and ΔSI truncations. In contrast, Aurora A was not detected in complex with H-Ras ΔSI&II. These data suggest that the N-terminal of H-Ras is necessary for the interaction with Aurora A since deletion of this region abrogates binding (Figure 2B). It appears that truncations containing the switch II region (36-66) showed positive interactions while removal of this region led to loss of Aurora A binding, which supports the importance of the 36-66 region in Aurora A interaction. However, whether the region of Ras containing amino acids 36-66 is sufficient for Aurora A binding requires further studies with refined fragments.

The N-terminal of H-Ras is highly conserved between the H-, K-, and N-Ras proteins. To test if Aurora A may also interact with other Ras proteins, we conducted a GST pull-down assay with the three predominant Ras isoforms found in cancer. Indeed, binding of Aurora A was detected with K-Ras and N-Ras as well as H-Ras (Figure 2C), suggesting that Aurora A may be able to engage Ras isoforms in cancer types driven by expression of any of the three major isoforms.

### The kinase domain of Aurora A mediates the H-Ras interaction

To characterize the domains of Aurora A that mediate binding to H-Ras, truncations of Aurora A were generated and tested for binding by GST pull-down. Venus-Flag Aurora A truncations are shown in Figure 2D: the N-terminal and kinase domains of Aurora A (NK, amino acids 1-383), the N-terminal fragment of Aurora A (N, amino acids 1-130), the kinase domain alone (K, amino acids 130-383), and the C-terminal domain (C, amino acids 383-403). Full length Aurora A binds to H-Ras, but not to GST (Figure 2E). Binding of the NK and K truncations of Aurora A to H-Ras was detected. Conversely, no binding of Aurora A truncations lacking the kinase domain (N and C) to H-Ras was observed. This binding pattern suggests that the region of Aurora A that interacts with H-Ras lies within the kinase domain of Aurora A.

Aurora B is an isoform of Aurora A that is also linked to cancer and can enhance the transformation of fibroblasts with the H-Ras G12V mutation [38]. The kinase domains of Aurora A and Aurora B are 53% homologous [19]. To determine if Aurora B is also able to bind to H-Ras, we conducted a GST pull-down assay to test their interaction. Indeed, Aurora B was capable of binding to H-Ras (Figure 2F).

### Aurora A enhances ERK phosphorylation

**Figure 3:**
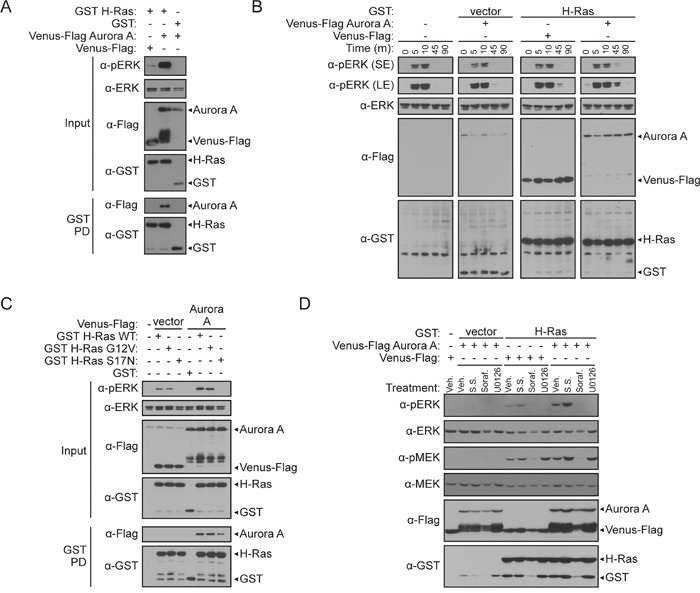
Aurora A potentiates ERK activation via H-Ras **A**. Detection of the Aurora A/H-Ras interaction correlates with enhanced pERK. GST pull-down (described in Figure 1B) between GST H-Ras and Venus-Flag Aurora A with corresponding western blot analysis of cell lysate inputs to assess changes in pERK compared to total ERK 48-hours post-transfection in HEK 293T cells. **B**. Aurora A sustains pERK levels in MCF-7 breast cancer cells. MCF-7 cells were either untransfected, transfected with Venus-Flag Aurora A or GST H-Ras with appropriate controls, or transfected with GST H-Ras and Venus-Flag Aurora A. As detected by western blotting, changes in pERK induced by co-transfected plasmids was assessed after cells were stimulated with serum for 0, 5, 10, 45, and 90 minutes after 24-hours of serum starvation. A short exposure (SE) and longer exposure (LE) of pERK is shown. **C**. H-Ras activity is required for potentiation of pERK by Aurora A. GST pull-down comparing binding and signaling changes between co-expression of GST H-Ras (WT), GST H-Ras G12V activating mutant, or GST H-Ras S17N dominant negative mutant with Venus-Flag Aurora A in HEK 293T cells. Western blot analysis of inputs to assess changes in pERK compared to total ERK 48 hours post-transfection. **D**. Use of a pharmacological probe for the MAPK signaling pathway in HEK 293T cells co-expressing Aurora A and H-Ras alone or in combination. 24-hours post transfection, cells were treated with DMSO vehicle control (Veh.), serum starvation (S.S.) Sorafenib (Soraf.) or U0126 at 10 μm then subjected to a GST pull-down and western blot analysis. Western blotting was conducted using anti-Flag, anti-GST, anti-pMEK, anti-MEK, anti-pERK, and anti-ERK antibodies.

Aurora A interacts with a region of H-Ras that mediates effector engagement and oncogenic signaling. Downstream from Ras proteins, MAPK signaling is a critical pathway for sustained proliferative signaling in many cancers. Therefore, we sought to examine the functional impact of the Aurora A/H-Ras interaction on the MAPK pathway. We first used western blotting to evaluate the impact of co-expressed Aurora A and H-Ras on ERK phosphorylation as a readout for MAPK signaling. As shown in Figure 3A, no detectable effect on ERK phosphorylation was observed when Aurora A was expressed alone, while the expression of H-Ras alone induced ERK phosphorylation. Interestingly, co-expression of Aurora A and H-Ras further enhanced ERK phosphorylation compared to H-Ras alone. By conducting a GST pull-down in parallel, we confirmed that the observed increase in ERK phosphorylation correlated with the interaction of Aurora A and H-Ras (Figure 3A).

Since Aurora A enhanced ERK phosphorylation when co-expressed with H-Ras in HEK 293T cells, we next sought to determine if Aurora A also affected ERK phosphorylation in cancer cells. With the sustained activation that occurs in cancer, ERK translocates to the nucleus to promote the transcription of genes that drive cell cycle progression [39, 40]. Therefore, we also tested if Aurora A sustains ERK phosphorylation in a temporal, serum-dependent manner.

To do this, we utilized breast adenocarcinoma-derived MCF7 cells. Aurora A has been previously investigated as a therapeutic target in breast cancer and overexpression of Aurora A and robust ERK levels occur in this cell line [41]. Our results show that in conditions without H-Ras expression, serum starvation blocks ERK phosphorylation and serum stimulation induces ERK phosphorylation in a temporal manner (Figure 3B). However, in serum starved cells expressing H-Ras, ERK phosphorylation levels are elevated in the presence of Aurora A compared to the vector control (Figure 3B, lane one of panels three and four). Lastly, after serum release, Aurora A prolongs ERK activation when co-expressed with H-Ras compared to expression of H-Ras alone (Figure 3B, panels three and four).

Together, these data show that the co-expression of Aurora A and H-Ras enhances and sustains ERK phosphorylation.

### Aurora A-induced ERK phosphorylation requires Ras-MAPK signaling

To clarify if the enhanced ERK phosphorylation observed in the presence of Aurora A and H-Ras requires Ras-MAPK signaling, we first employed site-specific inactivating or activating H-Ras mutants [42]. An activating mutant that mimics GTP-binding (GST H-Ras G12V) and a dominant negative GDP-binding preferred mutant (GST H-Ras S17N) were tested for the ability to interact with Venus-Flag Aurora A by GST pull-down in HEK 293T cells. When H-Ras WT or G12V were expressed in cells, ERK phosphorylation was stimulated (Figure 3C). In contrast, H-Ras S17N effectively blocked ERK phosphorylation. Co-expression of Aurora A potentiated ERK phosphorylation in the presence of H-Ras WT and G12V, but not H-Ras S17N. In the GST pull-down, we observed that although Aurora A requires active H-Ras to potentiate ERK phosphorylation, Aurora A was able to bind the WT, G12V, and S17N forms of H-Ras (Figure 3C). These data also suggest that the activity and conformation of H-Ras minimally impacts the ability of Aurora A to bind H-Ras; however, increased ERK phosphorylation requires active H-Ras.

To further validate that Ras-MAPK signaling is required for ERK phosphorylation, we took an alternative approach, employing pharmacological inhibitors to probe the involvement of Raf-1 and MEK in the effect of Aurora A on ERK phosphorylation. If Aurora A acts through MAPK signaling to activate ERK, pharmacological inhibition of the MAPK pathway would block this effect. Following Aurora A and H-Ras co-expression in HEK 293Ts, cells were treated with Raf-1 and MEK kinase inhibitors. While expression of H-Ras was able to induce MEK and ERK phosphorylation in DMSO-treated cells, inhibition of Raf-1 and MEK by Sorafenib and U0126, respectively, inhibited ERK phosphorylation (Figure 3D). We then tested if the ERK phosphorylation triggered by Aurora A co-expression also requires active Raf-1 and MEK. Indeed, these inhibitors were able to block ERK phosphorylation induced by Aurora A. In this model, serum starvation was unable to reduce ERK phosphorylation [43]. Collectively, these results support the hypothesis that Aurora A potentiates ERK phosphorylation through the Ras-MAPK signaling.

### Aurora A forms a protein complex with H-Ras and Raf-1 and acts through H-Ras to enhance MAPK signaling

**Figure 4:**
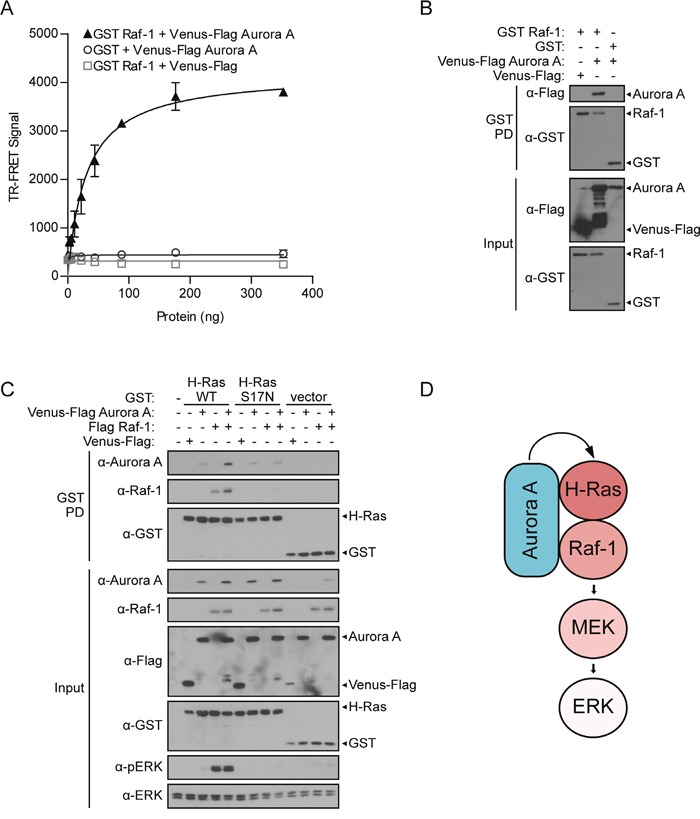
Aurora A forms a complex with H-Ras and Raf-1, acting through H-Ras to enhance ERK activation **A**. Aurora A directly interacts with Raf-1. TR-FRET was performed using HEK 293T lysates in which GST Raf-1 and Venus-Flag Aurora A along with vector controls were co-expressed. TR-FRET signals were recorded using an EnVision multilabel plate reader. Data shown are average signals with SD from duplicate samples. **B**. Aurora A associates with Raf-1. GST pull-down (as described in Figure 1B) between GST Raf-1 and Venus-Flag Aurora A with corresponding western blot analysis of inputs to assess changes in pERK compared to total ERK 48-hours post-transfection in HEK 293T cells. **C**. Aurora A/H-Ras/Raf-1 interactions stabilize the protein signaling complex. GST pull-down comparing the ability of wild-type (H-Ras WT) or dominant negative (H-Ras S17N) H-Ras to isolate either co-expressed Aurora A, Raf-1, or both proteins. Western blot analysis demonstrates binding of Aurora A or Raf-1 to H-Ras and the induced effect on pERK. Since both epitope-tagged proteins resolve around the same size, anti-Aurora A and anti-Raf-1 antibodies were used instead of anti-Flag. GST-tagged H-Ras WT and H-Ras S17N were detected using anti-GST antibody. Changes in pERK were detected using anti-pERK antibody. D. Proposed model for the role of Aurora A in the Aurora A/H-Ras/Raf-1 oncogenic singaling complex.

To initiate MAPK signaling, GTP-bound Ras must recruit Raf-1 to the plasma membrane to dimerize, transphosphorylate, and initiate the kinase cascade. As we have demonstrated that Aurora A engaged with the N-terminal domain of H-Ras that contains the effector binding domain and enhances MAPK signaling, we next sought to determine if Aurora A also associated with the Ras effector, Raf-1. TR-FRET results in HEK 293T cells showed that GST Raf-1 and Venus-Flag Aurora A exhibit a dose-dependent increase in TR-FRET signal compared to the negative controls (Figure 4A), providing evidence of an interaction of Aurora A with Raf-1. The binding of Aurora A and Raf-1 was also confirmed by GST pull-down (Figure 4B).

Since Aurora A interacts with both H-Ras and Raf-1, one mechanism by which Aurora A may enhance Ras-MAPK signaling is by stabilizing the H-Ras/Raf-1 protein complex. To test this hypothesis, we conducted a GST pull-down assay testing the binding of both Venus-Flag Aurora A and Flag Raf-1 to either GST H-Ras WT or GST H-Ras S17N. Results from HEK 293T cells revealed that H-Ras WT forms a protein complex with Aurora A and Raf-1 (Figure 4C). In addition, binding of both Aurora and Raf-1 to H-Ras WT is enhanced and ERK phosphorylation is strongly increased when all three proteins are co-expressed. In contrast to H-Ras WT, Raf-1 does not bind H-Ras S17N. Further, this inactive H-Ras mutant maintains the ability to interact with Aurora A, but Aurora A/H-Ras S17N binding was not enhanced as is observed with H-Ras WT.

An assessment of signaling changes demonstrates that ERK phosphorylation levels are tightly linked to Aurora A/H-Ras/Raf-1 protein complex formation. Aurora A further enhances the ERK phosphorylation stimulated by H-Ras or Raf-1. Further, ERK remains inactive when Aurora A or Raf-1 are expressed with H-Ras S17N (Figure 4C).

Together, these data demonstrate that Aurora A forms a protein complex with both H-Ras and Raf-1, stabilizes the H-Ras/Raf-1 interaction, and promotes MAPK signaling (Figure 4D). We also reveal that although Aurora A interacts with both H-Ras and Raf-1, H-Ras activity is required for the ability of Aurora A to enhance MAPK signaling.

## DISCUSSION

The family of Ras proteins (H, K, and N-Ras) function as oncogenic drivers in many cancer types by transmitting pro-growth and proliferative signals through the Ras-MAPK pathway. In this study, we identify a novel protein-protein interaction between Aurora A and Ras that provides a mechanism by which Aurora A acts as a positive regulator of Ras-MAPK signaling [32–35].

Using complementary protein-protein interaction assays, we demonstrated that Aurora A interacts with H-Ras and Raf-1, functioning upstream of Ras in the MAPK pathway to potentiate Ras-mediated MAPK signaling. Cooperation between Aurora A and Ras-MAPK signaling pathway is implicated in various cancer models. For example, Aurora A overexpression and Ras alterations co-occur in pancreatic, colon, and bladder cancers [32–35]. Additionally, modulation of ERK activity and the ETS promoter alters Aurora A expression, indicating that MAPK signaling regulates transcription of Aurora A [18]. Other studies place Aurora A upstream of MAPK signaling, enhancing H-Ras G12V transformation [35, 44] Similarly, knockdown of Aurora A in nasopharyngeal cancer cells reduced invasion by reducing activation of Ras pathway components [36]. Our work, taken together with independent studies by others, suggests that Aurora A may form a positive feedback loop that contributes to cell growth and proliferation [18, 34, 35, 38, 44–53].

In characterizing the structural domains that mediate the Aurora A/H-Ras interaction, we identified that a region within the kinase domain of Aurora A (amino acids 130 – 383) interacts within the N-terminal domain of H-Ras (amino acids 1-66). Since Aurora A binding is maintained with the deletion of the switch I domain of H-Ras, these data suggest that the required Aurora A binding site on Ras may be narrowed to amino acids 36-66, near the switch II domain of H-Ras.

Although most of our characterization was conducted with H-Ras, the demonstrated interaction of Aurora A with the three isoforms of Ras suggests a broad role of Aurora A in regulating the Ras-MAPK signaling mediated by isoforms of Ras in various cellular environment (Figure 2C). Considering that gain-of-function Ras alterations drive 30% of cancers, the ability for Aurora A to interact with these Ras isoforms provides a basis for the general impact of Aurora A overexpression in cancer. Our data support a role of Aurora A in enhancing the Ras signaling, possibly also on K-Ras and N-Ras beyond H-Ras, which may trigger positive regulatory circuits through induced Aurora A expression. This regulatory circuit may serve as a potential target for therapeutic exploitations in cancers of different tissue types, such as K-Ras-driven lung cancers and N-Ras-driven melanoma. The observed requirement of active Ras for Aurora A-triggered ERK pathway stimulation implies a potential cancer cell specific event mediated by the Aurora A/Ras connectivity (Figure 3C). It is tempting to speculate that such an Aurora A/Ras interface may be particularly vulnerable in tumors driven by activating mutant Ras. Thus, the Aurora A/Ras connectivity may offer a new targeting site for future therapeutic discovery.

Our finding that Aurora A interacts with Ras isoforms adds to previous reports of binding between Aurora family proteins and other GTPases and Ras-binding proteins. Aurora A interacts with RalA [47], Aurora A and B bind Ras GAP [54, 55], and Aurora B binds MgcRacGAP [56]. Both Aurora A and B have been implicated in cancer, thus the confirmation that Aurora B is also able to interact with H-Ras also expands the implications of this work and compliments studies in which Aurora B was found to associate with Survivin and RasGAP, and to stabilize Ras expression [53]. At this state, it remains unclear if Aurora B interacts with H-Ras while in complex with Survivin, or independently.

Raf-1 was identified to associate with Aurora A and other cell cycle machinery during mitosis [57, 58]. This led to the idea that Raf-1 may also exert MAPK-independent roles. Our finding reveals that Aurora A forms a protein complex with H-Ras and Raf-1, also placing the Aurora A/Raf-1 interaction in the context of Ras-MAPK signaling. The association of Aurora A with H-Ras does not appear to compete with H-Ras/Raf-1 binding and in fact, enhances the protein complex. Since the Aurora A/H-Ras/Raf-1 complex does not form and MAPK signaling is not stimulated without active H-Ras, we were also able to show that H-Ras activity is required for Aurora A-induced Ras-MAPK signaling.

Beyond the interactions we discovered, how Aurora A leads to enhanced Ras-MAPK signaling remains to be established. It is possible that the Aurora A/H-Ras interaction may increase GEF activity, prevent GAP activity, or induce an active conformation of H-Ras. Ras G12V is a mutant of Ras that binds GAP but is unable to hydrolyze GTP. Because we were able to demonstrate that co-expression of Aurora A and H-Ras G12V also enhances ERK activation, the mechanism of action of Aurora A may be Ras-GAP independent despite reports that both Aurora A and Aurora B both associate with Ras GAP [55, 59].

Attempts to directly target Ras proteins for cancer treatment have been largely unsuccessful in the clinic [59, 60]. Another opportunity to inhibit Ras signaling is by targeting protein-protein interactions that affect the regulation of Ras. Therefore, our identification of the novel interaction between Aurora A and H-Ras as a mechanism by which Aurora A can activate Ras-MAPK signaling opens the way for studies into perturbation of the Aurora A/H-Ras interaction and the effect on Ras-MAPK signaling. Evidence from these future studies would suggest that the interactions between Aurora A and Ras may serve as a therapeutic target in cancer.

## MATERIALS AND METHODS

### Cell culture

HEK 293T and MCF7 cells were utilized in the described experiments (American Type Culture Collection, Manassas, VA). HEK 293T and MCF7 cells were cultured in DMEM (Corning, MT10013CV, Manassas, VA) with 10% FBS (Sigma, F6178, St. Louis, MO) and 1% pen/strep at 5000 I.U/ml penicillin and 5000 μg/ml streptomycin (Corning, 30-001-Cl, Manassas, VA). Between passages, cells were trypsinized with 0.25% Trypsin with 2.21 mM EDTA (Corning, 25-053-Cl, Manassas, VA). All cells were maintained at 37°C in a humidified atmosphere of 5% CO2.

### Antibodies

Primary antibodies used for western blotting include Flag M2 at 1:3000 (Sigma, F3165, St. Louis, MO), Flag-HRP at 1:1000 (Sigma, A8592, St. Louis, MO), GST Z-5 at 1:3000 (Santa Cruz, sc-459, Dallas, TX), rabbit GST-HRP at 1:1000 (Sigma, A7340, St. Louis, MO), Aurora kinase A at 1:500 (Cell Signaling, 4718, Boston, MA), rabbit pERK and ERK (Cell Signaling, 4370, 9102, respectively, Boston, MA), pMEK and MEK (Cell Signaling, 9154, 4694, respectively, Boston, MA), pRaf-1 (Cell Signaling, 9427, Boston, MA) and Raf-1 (Santa Cruz, sc-133, Dallas, TX) at 1:1000, and Ras (BD Transduction Laboratories, 610002, San Jose, CA) at 1:1000. Secondary antibodies include goat anti-rabbit IgG (Santa Cruz, sc-2004, Dallas, TX) and goat anti-mouse IgG (Santa Cruz, sc-2005, Dallas, TX) and were used at either 1:2500 or 1:5000 dilutions. Antibodies used for immunoprecipitation include Aurora A (Sigma, A1231, St. Louis, MO), Ras (ThermoScientific, MA1-012X, Waltham, MA), and IgG (Santa Cruz, sc-2027, Dallas, TX) at 1:50 dilution.

### Pharmacological inhibitors

Sorafenib p-Toluenesulfonate Salt (S-8502) and U0126 (U-6770) inhibitors were obtained from LC Laboratories (Woburn, MA). Compounds were dissolved in dimethyl sulfoxide (DMSO) as 10 mM stock and stored at −20°C. Cells were treated for 24 hours with 10 μM of compounds diluted in DMSO.

### Serum starvation

MCF7 cells were plated in a 24-well plate at 1×10^5^ cells per well and cultured in 600 μl of complete medium (as described, DMEM with 10% FBS and 1% Penicillin/Streptomycin). Cells were then transfected 24 hours after plating. Complete media was replaced with DMEM media without FBS supplementation (serum free media) 24 hours after transfection. Samples were collected for the 0 minutes-post serum stimulation time point following 24 hours in serum-free media. Then, serum (10% FBS) was added to all remaining wells. Remaining cells were collected at time points 5, 10, 15, 45, and 90 minutes after serum stimulation. Cells were collected directly into 1X SDS loading buffer, boiled for 5 minutes, and subjected to SDS-PAGE and western blotting.

### Transfections

For experiments with ectopically expressed proteins, HEK 293Ts were transfected using X-tremeGENE (Roche, 06366546001, Basel, Switzerland). MCF7 cells were transfected with FugeneHD (Promega, E2312, Madison WI). Plated cells were transfected at a density of 60-80% confluency and performed with a ratio of 3 μl transfection reagent to 1 μg DNA to 100 μl of serum-free media. DNA was mixed at appropriate concentrations prior to the addition of serum-free DMEM. Transfection reagent was then added and incubated at room temperature for 15 and 20 minutes (X-tremeGENE and FugeneHD, respectively). Transfection complexes were then added drop-wise to plated cells.

### Plasmid construction

All plasmids of full length and truncated proteins were constructed using Gateway® technology (Invitrogen, Waltham, MA) per the manufacturer's protocols. For GST-tagged and Venus-Flag tagged plasmids used for Time Resolved-Föster Resonance Energy Transfer (TR-FRET) and glutathione-S-transferase (GST) pull-downs, pDEST27 and pFUW vectors were used as destination cloning vectors, respectively. Amino (N-Venus) and carboxy (C-Venus) plasmids used for Venus protein-fragment complementation assay (PCA) were generated previously in the lab. Aurora A or H-Ras cDNA was PCR amplified and inserted into the pDONR201 (Invitrogen) vector using a BP reaction to generate entry cloning vectors. A LR reaction was used to clone the desired DNA into the appropriate destination vectors. Constructs were verified by restriction digest using BSRGI (NEB, R0575L, Ipswich, MA) or FastDigest Bsp1407I (ThermoScientific, FD0933, Waltham, MA), both cutting at the T^GTACA attB1 and attB2 (entry clone) or attR1 and attR2 (destination vector) recombination sites, and DNA sequencing. Clones in pDEST-27 (GST) vectors were sequenced with forward primer 5′-AAGCCACGTTTGGTGGTG-3′ and the standard T7 reverse primer. Clones in pFUW (Venus-Flag) vectors were sequenced with forward primer 5′-CGATCACATGGTCCTGCTG-3′ and the standard SP6 reverse primer.

### Site-directed mutagenesis

Site-directed mutagenesis was performed on the GST H-Ras vector to create the catalytically-inactive mutant (S17N) using the QuikChange™ Site-Directed Mutagenesis Kit according to the manufacturer's protocol (Agilent Technologies, 210519, Santa Clara, CA). The H-Ras S17N mutant was generated using the oligonucleotide forward primer 5′-GGCGGTGTGGGCAAGAATGCGCTGACCATC-3′ and reverse primer 5′-GATGGTCAGCGCATTCTTGCCCACACCGCC-3′. Successful mutagenesis was confirmed by DNA sequencing as described previously.

### Protein-protein interaction studies

#### TR-FRET assay

TR-FRET was performed in 384-well black solid bottom plates (Corning Costar, 3654, Manassas, VA) in a total volume of 30 μL in each well. Briefly, HEK 293T cells were transfected as described above. Cells were lysed using 0.5% NP-40 lysis buffer (0.5% NP-40, 150 mM NaCl, 10 mM HEPES, and Phosphatase Inhibitor Cocktail (Sigma, P5726, St. Louis, MO) and Protease Inhibitor Cocktail (Sigma, P8340, St. Louis, MO)). Lysates were collected and centrifuged at 13,500 g for 10 minutes at 4°C to remove cellular debris. Cleared cell lysates were serially diluted in FRET buffer (20 mM Tris, pH 7.0, 0.01% Nonidet-P40, and 50 mM NaCl) in a 384-well plate, bringing the final volume of diluted cell lysate to 15 μL per well. Then, 15 μL of diluted anti-GST-Terbium antibody (Cisbio US Inc, 61GSTTLB, Bedford, MA) was added to all wells at a final dilution of 1:1000. The TR-FRET signals were detected with an EnVision Multilabel plate reader (PerkinElmer) with laser excitation at 337 nm, emission1 at 486 nm and emission2 at 520 nm. TR-FRET signal is expressed as ratio and calculated by the following equation: TR-FRET signal = F520/F486 × 10^4^, where F486 and F520 are fluorescence counts at 520 nm and 486 nm for Venus and terbium emission signal, respectively. Data were presented as mean with standard deviation calculated from duplicate samples.

#### GST pull-down

Cells were seeded in to a 6-well plate and allowed to reach 60-80% confluency. Cells were then harvested by adding 200 μL of 0.5% NP-40 lysis buffer to each well, collected by scraping, transferred to an eppendorf tube, and incubated at 4°C for 30 minutes. Lysis buffer components consisted of 0.5% NP-40, 150 mM NaCl, 10 mM HEPES lysis buffer, and Phosphatase Inhibitor Cocktail (Sigma, P5726, St. Louis, MO) and Protease Inhibitor Cocktail (Sigma, P8340, St. Louis, MO) at 1:1000. After incubation, lysates were centrifuged to remove cellular debris. After removing 20 μl of the lysate for an input control and the debris pellet, 20 μl of a 50% glutathione-conjugated sepharose bead slurry (Glutathione Sepharose 4B, Fisher Scientific, 50197956, Atlanta, GA) was added to the remaining lysate and incubated by slowly rotating for 3-4 hours at 4°C. Beads were then washed three times in 0.5% NP-40 lysis buffer by inverting 8 times with 200 μl of fresh lysis buffer added each time. GST-bound protein complexes were then eluted by the addition of 20 μl of 2x SDS loading buffer, boiled for 5 minutes, resolved by SDS-PAGE subjected to western blotting along with input controls.

#### Venus protein-fragment complementation assay

Cells were seeded into 24-well plates and transfected at 60% confluency with N-Venus or C-Venus constructs. After 24 hours, cell nuclei were stained with the addition of Hoechst 33342 (Fisher Scientific, H1399, Atlanta, GA) at 5 μg/ml. Images were then acquired using the ImageXpress^Micro^ automated imaging high-content imaging system (Molecular Devices) with 20X objective. The standard filter set for FITC (excitation 482/35 nm and emission 536/40 nm) and DAPI (excitation 337/50 nm and emission 447/60 nm) was used for Venus and Hoechst 33342 imaging, respectively. The number of green (Venus) and total cells (Hoechst 33342) from the images were calculated using the Metamorph Analysis Cell Scoring module and presented as percent of Venus positive cells compared to the total number of cells.

#### Co-immunoprecipitation

Cells were seeded in 15 cm dishes and grown to confluency. Cells were then washed with PBS and lysed in 0.5% NP-40 lysis buffer containing protease inhibitors (as previously mentioned), then incubated on ice for 30 minutes. Lysates were then centrifuged for clarification at 4°C. To preclear, protein A/G beads (Santa Cruz, sc-2003, Dallas, TX) were added and rotated with lysates for 30 minutes at 4°C. Cleared lysates were then transferred to a new tube for immunoprecipitation where they were then incubated with antibodies against Aurora A, Ras, or IgG at 1:50 dilution for 16 hours at 4°C, followed by adding 25 μl of protein A/G beads and rotating for an additional 8 hours. Aurora A or Ras immunocomplexes on beads were washed three times with 0.5% NP-40 lysis buffer containing protease inhibitors and eluted into 2X SDS loading buffer prior to SDS-PAGE and western blot analysis.

#### Western blotting

Cell lysates were subjected to western blot analysis following protein separation by SDS-PAGE (10% acrylamide gels) and subsequent transfer to PVDF membranes at 100V for 1.5 hours. Membranes were blocked in TBST (50 mM Tris, 137 mM NaCl, 0.05% Tween, pH 7.6) containing 5% dry milk for 30 minutes – 1 hour at ambient temperature, then incubated at 4°C or ambient temperatures with primary antibodies diluted in 5% milk in TBST. After primary antibody incubation, membranes were washed three times with TBST for 5 minutes each, then incubated with secondary antibody for 1 hour at ambient temperatures. For HRP conjugated antibodies, membranes were blocked with milk, then washed three times with TBST for 10 minutes each, then incubated with GST-HRP or Flag-HRP for 1 hour. Membranes were then washed three times with TBST for 10 minutes each and chemiluminescent signal (West Pico, West Dura (ThermoScientific, PI34080 or PI34076, respectively, Waltham, MA) or ECL (Amersham, 84-839, San Diego, CA) was added for 5 minutes prior to developing by autoradiography. Proteins with the Venus-Flag epitope tag were detected by blotting with anti-Flag antibody.
